# Dynamic MRI of the fetal myocardium

**DOI:** 10.1186/1532-429X-14-S1-P236

**Published:** 2012-02-01

**Authors:** Christopher W Roy, Mike Seed, Joshua van Amerom, Lars Grosse-Wortmann, Shi-Joon Yoo, Chris Macgowan

**Affiliations:** 1 Medical Biophysics and Medical Imaging, University of Toronto, Toronto, ON, Canada; 2 Divison of Cardiology, Department of Paediatrics, The Hospital for Sick Children, Toronto, ON, Canada; 3 Diagnostic Imaging, The Hospital for Sick Children, Toronto, ON, Canada

## Background

Fetal cardiovascular MRI has been hampered by the lack of a reliable cardiac gating signal. A recently proposed solution to this problem is metric optimized gating (MOG) [1-3]. Here, we demonstrate the ability of MOG to acquire images of the fetal myocardium without conventional cardiac gating. Our work is motivated by the need for high-resolution dynamic imaging in the assessment of fetal congenital heart disease [4].

## Methods

Fetal scans were performed using a 1.5T Avanto MRI system (Siemens, Germany). Scan lengths were kept as short as possible to avoid artifact from fetal movement (~ 5-10 seconds/slice). Data were acquired using a conventional cine pulse sequence triggered by a synthetic cardiac gating signal. The period of this trigger was constant, and chosen to be longer than the expected duration of the fetal cardiac cycle. This ensured that each line of k-space was acquired for every cardiac phase. Data were then retrospectively sorted and reconstructed using hypothetical cardiac triggers. The positions of these triggers were iteratively adjusted according to the MOG method until a metric for image quality (entropy) was optimized [1,2,5].

## Results

**Figure 1:**
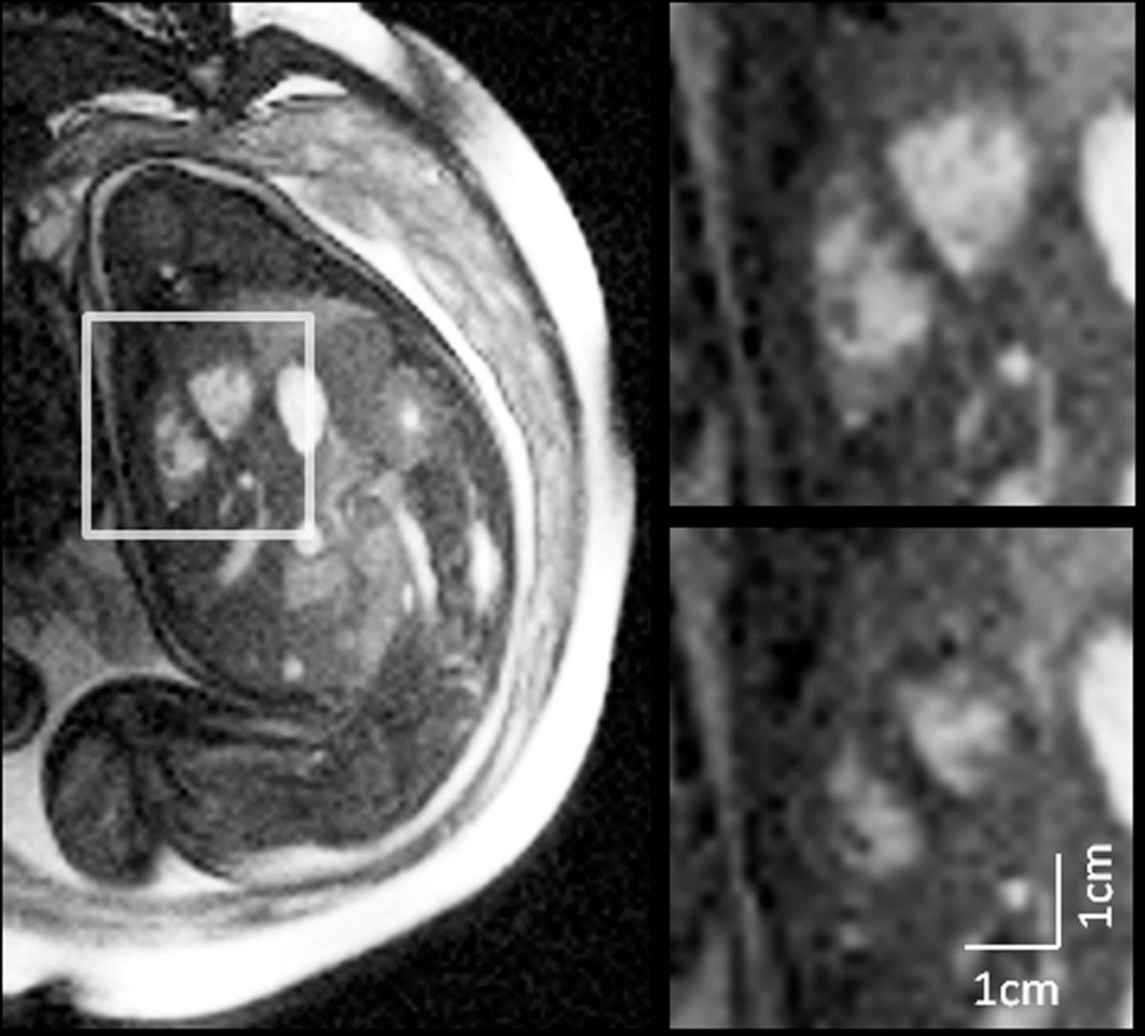
Short axis images of the fetal heart reconstructed using MOG. The full field of view (left) as well as diastolic (top right) and systolic (bottom right) images are shown for comparison. Scan parameters were as follows: TR/TE = 3.1/1.2 ms, FA = 70°, matrix = 240x176, voxel = 1.3x1.3x5 mm3, views-per-segment = 15, scan length = 8 s.

**Figure 2:**
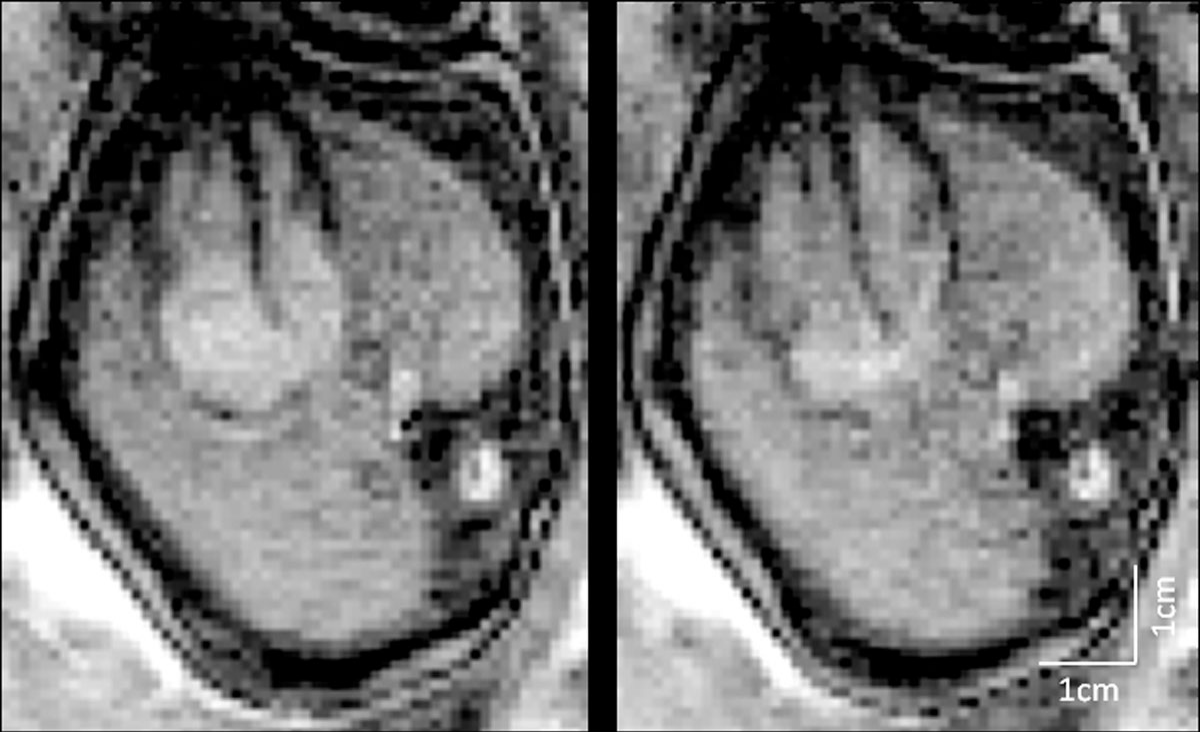
Four chamber view of the fetal heart reconstructed using MOG. Systolic (left) and Diastolic (right) images are shown for comparison. Scan parameters were as follows: TR/TE = 3.1/1.2 ms, FA = 70°, matrix = 246x176, voxel = 1.3x1.3x5 mm3, views-per-segment = 15, GRAPPA acceleration factor =2, scan length = 5 s.

Figures 1 and 2 show short-axis and four-chamber views of the fetal heart, respectively. In Figure 1, the ventricles are shown at end-systole and end-diastole. Normal myocardial contraction is visible when comparing the two images and assessment of cardiac function is possible (LV ejection fraction=60%). Similarly, Figure 2 shows a four chamber view of the fetal heart at end-systole and end-diastole. Although the atrioventricular valves are better appreciated on the moving cine series, intracardiac structures can be identified in both figures. The short axis images at mid ventricular (Figure 1) level demonstrate the LV papillary muscles, the moderator band of the RV and show thickening of the myocardium in systole. In the four chamber view (Figure 2), longitudinal contraction of the heart can be appreciated by comparing the position of the atrioventricular groove on the systolic and diastolic images.

## Conclusions

Using MOG, MRI of the human fetal myocardium was possible despite the absence of conventional cardiac gating. We were able to identify moving structures of interest during radial (Figure 1) and longitudinal (Figure 2) contraction, thus capturing normal fetal myocardial motion and permitting assessment of cardiac function. Furthermore, visualization of these results as movie loops provided the location of several features of interest that would not be visible without synchronization to the underlying fetal cardiac cycle.

## Funding

Christopher Roy was supported through a studentship in part by the Ontario Opportunity Trust Fund - Hospital for Sick Children Foundation Student Scholarship Program and a Canadian Graduate Scholarship from the Natural Sciences and Engineering Research Council of Canada.
